# Improved nanoparticle productivity via flat-top 49-beam nanosecond pulsed laser ablation in liquids

**DOI:** 10.1186/s11671-026-04924-9

**Published:** 2026-09-16

**Authors:** Abdel Rahman Altakroury, Rémi Bérard, Oleksandr Gatsa, Jan Lino Kricke, Zongwen Fu, Carlos Doñate-Buendía, Alexander V. Bulgakov, Bilal Gökce

**Affiliations:** 1 Chair of Materials Science and Additive Manufacturing, School of Mechanical Engineering and Safety Engineering, University of Wuppertal, 42119 Wuppertal, Germany; 2 FZU - Institute of Physics of the Czech Academy of Sciences, Na Slovance 1999/2, Prague, 18200 Czech Republic; 3 GROC·UJI, Institute of New Imaging Technologies, Universitat Jaume I, Av. de Vicent Sos Baynat, s/n, Castellón, 12071 Spain

**Keywords:** Gold nanoparticles, Multiple beams, Investment-specific productivity, Diffractive optical element, Nanosecond pulsed laser ablation in liquid

## Abstract

Pulsed laser ablation in liquids (PLAL) is a versatile and environmentally friendly technique for producing surfactant-free, high-purity nanoparticles (NPs) suitable for applications in catalysis and biomedicine. However, its industrial scalability is limited by low throughput and the high cost of high-power ultrafast (ps/fs) laser systems typically required to achieve efficient NP production. Here, we demonstrate a cost-effective high-throughput PLAL approach using a high-power nanosecond laser (500 W) combined with a diffractive optical element (DOE) that splits each pulse into 49 identical sub-beams. This multi-beam configuration enables operation at optimal fluences and spot interpulse distances,, which may contribute to reducing shielding effects and improving the ablation efficiency. Using gold as a model material, we achieve a NP productivity of ~ 3.3 g h⁻¹. Notably, the investment-specific productivity reaches 13.2 mg (h·k€)⁻¹, exceeding that of ultrafast laser-based systems and demonstrating superior economic efficiency without compromising NP size. Possible shielding factors such as cavitation bubble dynamics, persistent bubbles, and NP accumulation in the beam path are discussed to identify optimal ablation conditions. These findings provide important insights into PLAL upscaling and establish multi-beam ns PLAL as a promising route for high-throughput NP production.

**Clinical trial registration:**

Not applicable.

**Figure:**
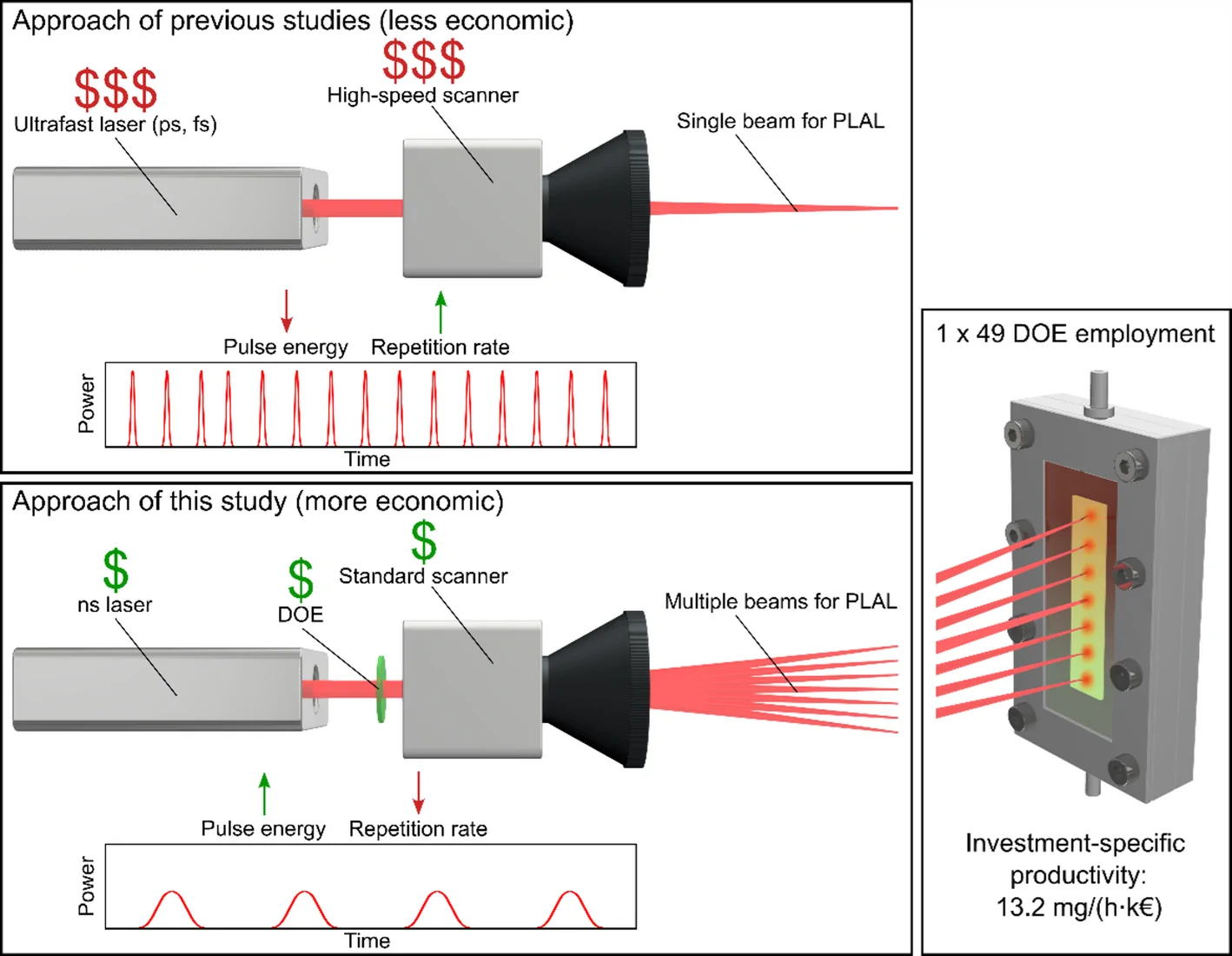

## Introduction

Pulsed Laser Ablation in Liquid (PLAL) is gaining attraction for the synthesis of nanoparticles (NPs) owing to its versatility to synthesise NPs from a wide range of materials [1, 2]. A key advantage of PLAL is direct generation of ligand-free, high-purity NPs, which are particularly attractive for industrial applications such as heterogeneous catalysis [3, 4] and biomedicine [5, 6], plasmonic sensing [7], as well as optoelectronics [8]. However, despite these advantages, the large-scale adoption of PLAL remains limited by relatively low productivity and the high cost of laser systems required for efficient NP generation.

PLAL relies on pulsed (femtoseconds, picoseconds, or nanoseconds) laser irradiation of a solid target immersed in a liquid medium. The absorbed laser energy induces vaporisation and ionisation of the target material, forming a hot transient plasma. The plasma is cooled by the surrounding liquid, leading to the nucleation of clusters, which subsequently yield NPs before being released into the liquid [1, 5]. An important feature of the PLAL process is the generation of a cavitation bubble, which can shield subsequent laser pulses, reducing the energy delivered to the target and thus limiting productivity [9, 10]. To mitigate this effect, bypassing the cavitation bubble is pivotal. Spatial bypassing by laser scanning or temporal bypassing by optimising the repetition rate [11] are two approaches implemented to minimise the cavitation bubble shielding and increase the spatial separation between two consecutive pulses on the target when a moving stage or scanning system is used.

In addition to the cavitation bubble effects, PLAL is governed by laser fluence and pulse duration. Above the material-specific ablation threshold fluence, the productivity typically increases with fluence until reaching an optimal regime [12, 13]. Beyond this optimum, productivity typically starts to decay due to different factors such as non-linear absorption [14], avalanche ionisation [15], and multiphoton absorption [16]. Such losses are more pronounced for shorter pulses of fs durations [17]. Fluence can also alter the cavitation bubble dynamics, where increasing fluence can increase cavitation bubble volume, which promotes beam shielding and ablation efficiency losses [18]. Increasing the fluence can increase the formation of persistent microbubbles, which are typically gas-filled bubbles that have a long lifetime (seconds to minutes) and remain near the target during PLAL [19]. Absorption, reflection, and scattering of the incident beam by the persistent microbubbles cause significant shielding and ablation efficiency losses [20].

Typical PLAL productivities remain in the mg/h range [21], and upscaling to g/h throughput requires specific high-power and high-repetition-rate lasers. Waag et al. achieved productivity of 8.3 g/h for Au-Pt alloy NPs using a high repetition-rate 500 W ps laser combined with a high-speed polygon scanner [22]. Such systems are, however, complex and costly, limiting industrial scalability. An alternative approach is developed by Khairani et al. [23] and Gatsa et al. [24] and is based on the use of diffractive optical elements (DOEs) to spatially split the laser beam into several sub-beams. With this method, a productivity of ~ 1 g/h was achieved for Au NPs using a 10-ps laser by employing a 1 × 11 beam DOE along with a galvanometer scanner [23], which is significantly cheaper than the polygon scanner. It was demonstrated that beam splitting allows using desirable fluence while lowering the repetition rate for multi-beam PLAL, thereby increasing the mass productivity due to the larger interpulse distance that reduces cavitation bubble shielding. Lower repetition rates could also be used to lower the probability of heat accumulation and enhance thermal diffusion while further increasing interpulse distance [25]. This defines the importance of DOE at a low repetition rate.

Despite these advances, the developed high-throughput PLAL approaches rely on picosecond laser systems, which remain significantly more expensive than nanosecond lasers. This cost difference primarily arises from the underlying pulse generation mechanisms. Mode-locked ps laser systems require precise phase control and high-damage-threshold optical components, whereas ns lasers are typically based on more robust and cost-effective Q-switching techniques [26, 27]. While ultrashort pulses offer advantages such as reduced thermal effects and so-called “cold ablation” [28, 29], Nanosecond laser ablation operates predominantly via photothermal processes governed by thermal diffusion, melting, and resolidification. Consequently, the material’s thermal properties heavily govern the ablation efficiency. In highly thermally conductive materials, rapid heat dissipation away from the focal volume reduces local thermal energy density, thereby increasing the effective ablation threshold. Conversely, in thermally insulating materials, heat remains localised at the surface, enabling lower ablation thresholds. Nevertheless, when operated at fluences sufficiently above the ablation threshold, nanosecond lasers remain effective for processing conductive materials [30]. Furthermore, for a given average power, ns lasers provide higher pulse energies, enabling greater flexibility in achieving optimal fluence conditions, particularly when combined with beam splitting.

In this study, we demonstrate a high-throughput and cost-effective PLAL approach by combining a high-pulse-energy ns laser with a DOE that splits the raw beam into 49 sub-beams. This configuration enables operation at optimal fluence while maintaining a low repetition rate (10 kHz) and a relatively large interpulse distance. Using gold due to its chemical nobility (resistance to in-situ oxidation during PLAL), high relevance in catalytic and biomedical applications, and widespread use as a benchmark material in PLAL upscaling studies, we show that this approach significantly enhances nanoparticle productivity and provides a scalable pathway toward economically viable industrial PLAL.

## Materials and methods

### PLAL setup

**Fig. 1:**
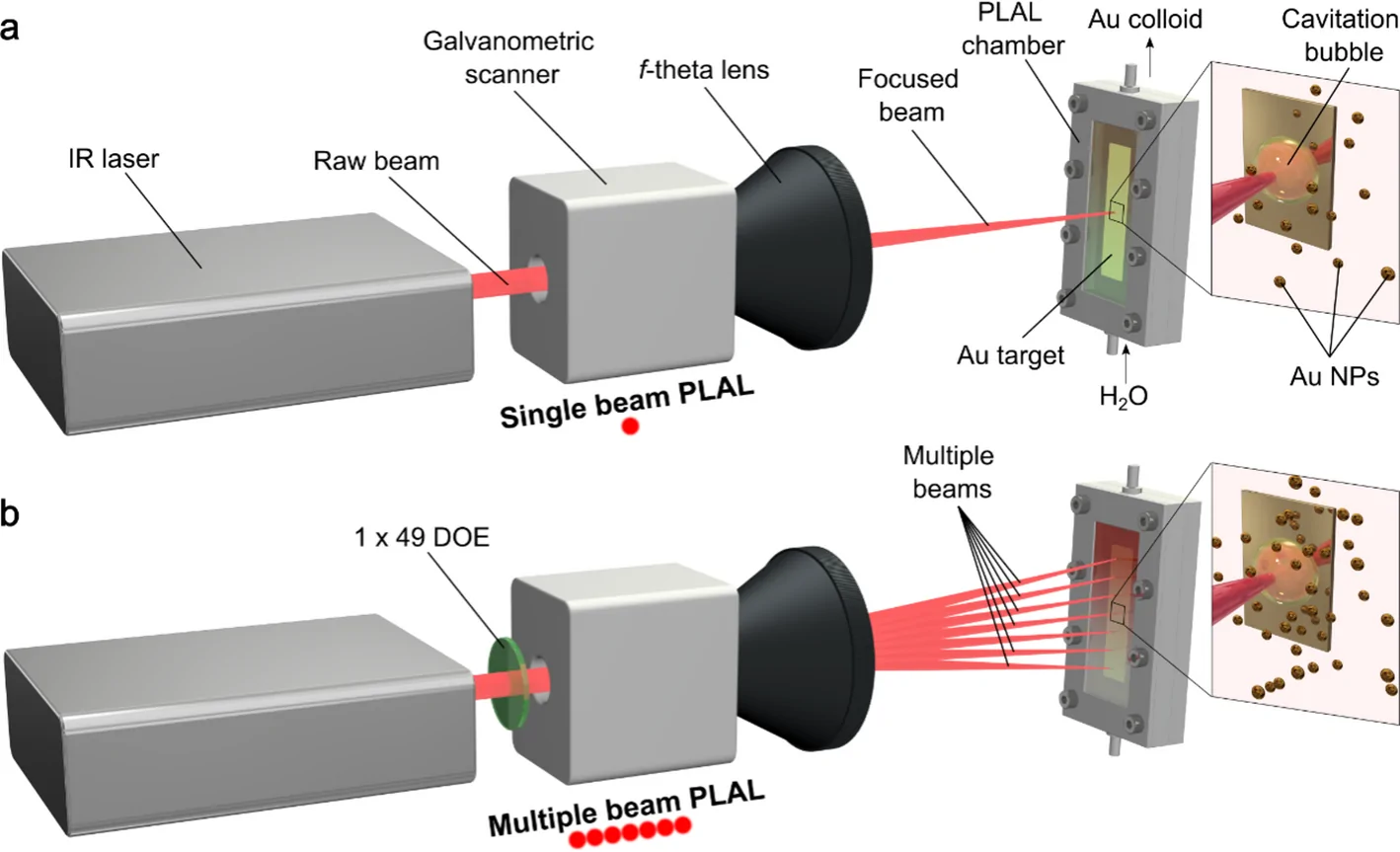
Schematic of the experimental setup illustrating the laser, the galvanometer scanner and the target installed inside the chamber. **a** single beam setup, and (**b**) multi-beam setup with the DOE installed

Figure 1 represents the PLAL experimental setup, where a high-power ns laser (Edgewave GmbH, Germany; pulse duration: 10 ns, average power up to 500 W, wavelength: 1064 nm) with manufacturer-specified beam quality factors of M_x_^2^ = 3.24 and M_y_^2^ = 4.92 were used. The raw output beam had dimensions of approximately 4 × 1 mm. To achieve a uniform intensity distribution, a 1D beam expander was used to expand the beam to 4 × 4 mm, followed by a 3 mm circular aperture to cut the profile, yielding a 3 mm circular flat-top beam. The incident pulse energy was varied from 200 µJ to 30 mJ, and the repetition rate was maintained at 10 kHz for all conditions. A galvanometer scanner (SUPERSCAN IV-15, Raylase GmbH, Germany) with a scanning speed of 10 m/s and a f-theta lens with a focal length of 100 mm was used for PLAL. The resulting interpulse distance was 1 mm. A hollow spiral pattern with an outer diameter of 10 mm, an inner diameter of 2 mm and an interlinear distance of 0.1 mm (spiral pitch) was used for all the experiments. Compared to the conventional raster scanning pattern, the spiral pattern avoids the deceleration, dwell, and acceleration delays that occur at line ends and turnaround regions during scanning. This ensures a consistent linear scanning velocity and uniform interpulse distance across the ablation area. Moreover, the spiral pattern has previously demonstrated high ablation efficiency in PLAL [23]. First, PLAL was performed using a single beam condition (without the DOE) to find the optimal ablation fluence and understand the trend of Au NP productivity for the setup as illustrated in Fig. 1 (a). Afterwards, a 1 × 49 DOE (UV-FS, 2-sided AR coating, Holo/Or, Israel) was installed just before the scanner to generate a line of 49 identical beams for irradiation as illustrated in Fig. 1 (b). The DOE shows very high transmittance close to 100%, and the splitting non-uniformity among its sub-beams is less than 3% (between the highest pulse energy beam and the lowest pulse energy beam). This guarantees an exceptionally uniform pulse energy distribution across all beams, enabling accurate calculation of individual beam fluence. A power meter (Plus2, measuring range of up to 500 W, LaserPoint SRL, Italy) was used for laser power characterisation after the galvanometer scanner. The power measurement after the scanner directly corresponds to the incident power on the target.

Au targets (99.999%, EVOCHEM Advanced Materials GmbH, Germany) with dimensions of 30 × 10 × 1 mm^3^ were used as PLAL targets for all the experiments. The target was placed vertically in an aluminium ablation chamber with internal dimensions of 85 × 30 × 10 mm^3^. A 2 mm quartz window with antireflective (AR) coating was used to cover the chamber. The thickness of the liquid layer (distance between the Au target and the quartz window) was maintained 9 mm in all cases. The chamber was connected with a peristaltic pump (LabV1, Shenchen Co., Ltd, China), with a water flow rate of 300 mL/min (ultrapure Mili-Q water with an electrical resistivity of 18.2 MΩ·cm at 25 °C, generated using a Synergy Water Purification System). The flow behaviour was set to be laminar with a linear velocity of ~ 16.7 mm/s inside the chamber, while complete water filling was maintained inside the chamber during the flow process. The flow rate was chosen to maximise NPs removal while maintaining stable laminar flow. Operating at ~ 60% of the pump’s maximum volumetric capacity (500 mL/min) avoids mechanical vibrations and micro-fluctuations that could induce local turbulence. Although a higher flow rate can contribute to increasing the efficiency of NPs removal from the irradiation region, operating pumps at full capacity should also be avoided. Thus, 300 mL/min provides a stable, consistent baseline for direct comparison across all experimental conditions.

**Fig. 2:**
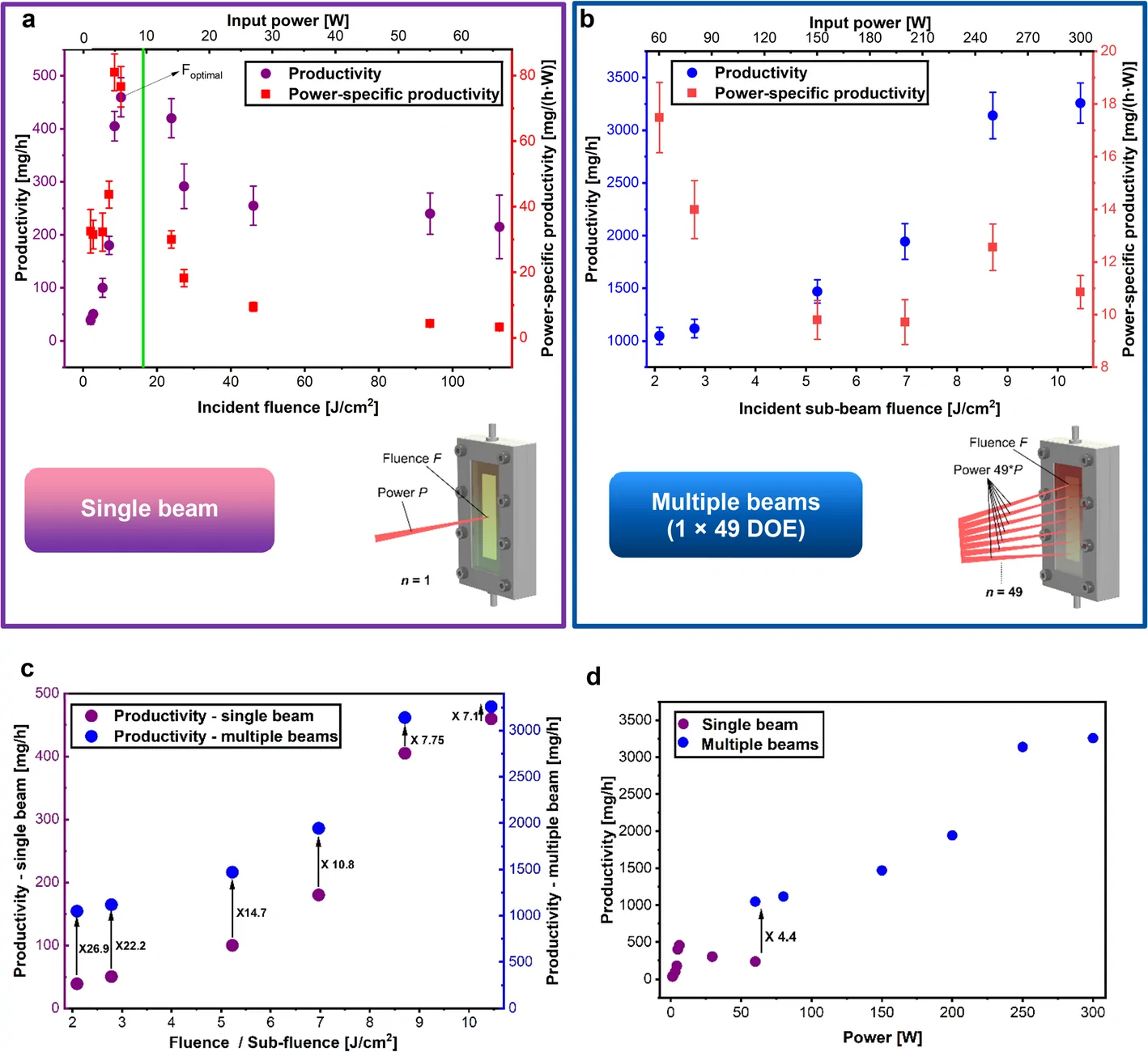
**a** Absolute and power-specific productivities vs. fluence (input power) for single-beam PLAL. The green line separates the increasing and decreasing parts of the dependencies. **b** Same as in (**a**) for multi-beam PLAL. Error bars in (**a**) and (**b**) represent the standard error of the mean (SEM) for *n* = 3 independent runs. **c** Productivity multiplicative factor of multi-beam to single-beam PLAL at different fluences. **d** Comparison of productivity of single-beam and multi-beam PLALs as a function of power, illustrating the productivity multiplicative factor for one representative condition at 60 W

The total ablation duration for each condition was set to 5 min. The average productivity was calculated from at least 3 measurements to ensure reproducibility and enable statistical analysis. All error bars in Fig. 2 represent the standard error of the mean (SEM) calculated from these replicates.

### Spot size determination

The spot size of the infrared laser with a flat-top beam profile after the f-theta lens was evaluated analytically from the Airy disk formula (1), where the raw beam is considered as a diffraction pattern and the aperture diameter represents the spot size. Moreover, the flat-top profile is considered a super-Gaussian profile with a high order number [31]. Then, with the beam diameter ($\:{D}_{0}$) of 3 mm, focal distance (* f*) of 100 mm, and the wavelength ($\:\lambda\:$) of 1064 nm, Eq. (1) gives the spot diameter of 86.5 μm.

1 $$\:{D}_{spot}\mathrm{=}2.44\cdot\:\lambda\:\cdot\:\frac{f}{{D}_{0}}\text{}$$

It should be noted that spot size calculation using the Airy Disk formula is considered an approximation. While experimental beam profiling (e.g., camera profiler) could yield slight variations in actual spot size and the corresponding fluence values. However, this theoretical estimation provides a consistent baseline for analysing and comparing productivity trends across the different conditions.

### Analysis (NP Productivity, NP size, DOE splitting)

The target weight was measured before and after ablation using an analytical weighing balance (Gram FS 220, Gram Group, Spain). After ablation, the samples were dried in an oven at 100 ^ᴼ^C to minimise the effect of moisture on the resulting weight. The difference between weight measurements corresponds to the 5-minute ablation mass productivity, which was then converted into productivity per hour (mg/h).

Scanning Transmission Electron Microscopy (STEM, ZEISS Gemini 460, maximum acceleration voltage of 30 kV, Germany) was used to analyse the produced NPs. Colloids containing Au NPs were drop-cast onto TEM grids (graphite / copper, 300 square mesh, Plano GmbH, Germany). The grids were placed on a weighing paper. Several drops were deposited to obtain a high population of NPs, and a hot plate was used to speed up the water evaporation.

An optical microscope (Leica DM2700 M, Germany) was used to evaluate the DOE splitting distance and total length of the DOE array, where the DOE beams induced 49 craters on a metallic target.

## Results and discussion

PLAL was carried out using a single laser beam at various fluence values (2 J/cm^2^ to 113 J/cm^2^). Evaluating how mass productivity depends on fluence provides a useful basis for understanding productivity trends and identifying the fluence that yields maximum productivity (F_optimal_). This single-beam fluence productivity relationship is then used to determine the appropriate fluence values for the individual sub-beams generated by the DOE (sub-beam fluence). It was found that fluence exceeding 113 J/cm^2^ lacks the benefit of increasing the productivity of Au NPs, thus further increase of the fluence is not necessary.

Figure 2 (a) illustrates the relationship between productivity in mg/h and power-specific productivity in mg/(h·W) in the single-beam regime, both as a function of fluence. At the lowest fluence of 2 J/cm^2^, a productivity of ~ 40 mg/h was obtained. Beyond 2 J/cm^2^, the productivity increases significantly until reaching ~ 460 mg/h at F_optimal_ ~10.5 J/cm^2^. Whereas, the highest power-specific productivity of 81 mg/(h·W), was obtained at a lower fluence of 8.5 J/cm^2^. A clear decrease in productivity is observed beyond F_optimal_; i.e., at values exceeding the green line in Fig. 2 (a).

This decrease is explained by the shielding factors, which reduce the energy absorbed by the target. Plasma and bubble dynamics are also greatly altered at high fluence, which can also produce further shielding and significantly decrease the productivity [32]. 

The fluence range from 2 to 10.5 J/cm^2^ (i.e., before the green line in Fig. 2 (a)) was selected for sub-beam fluences in the multi-beam PLAL. In the latter case, the input power was increased 49-fold (up to 300 W) to achieve an identical fluence per sub-beam compared to the single beam condition.

Figure 2 (b) illustrates productivity and power-specific productivity of multi-beam PLAL using 1 × 49 DOE.

With the DOE, productivity always increases with the input power up to the maximal used value of 300 W while it starts to decrease beyond 6 W when using the single beam. This allowed us to benefit from the available high power of the laser and to achieve the productivity of ~ 3250 mg/h at 10.5 J/cm^2^ sub-beam fluence in the multi-beam PLAL.

The power-specific productivity in the multi-beam regimes generally shows a decrease with increasing fluence. The highest value achieved is ~ 17.5 mg/(h·W) at the lowest sub-beam fluence of 2 J/cm^2^ (corresponding power 60 W, Fig. 2 (a)). This indicates the existence of shielding factors leading to losses in the ablation efficiency in the multi-beam PLAL, which increase with fluence as discussed below. Both Fig. 2 (a) and (b) illustrate relatively varied uncertainty seen visually by the standard error of the mean bars (SEM); this is caused by some factors such as pulse-to-pulse stability, weighing uncertainty, and slight changes in flow behaviour and the existence of micro-turbulence. Those factors can vary from one measuring experiment to another and cannot be completely avoided.

Figure 2 (c) illustrates the multiplicative factor of productivity between multi-beam and single-beam conditions at different fluences. At the same fluence, the multiplicative factor of productivity is expected to be close to 49 assuming a shielding-free environment. However, the highest multiplicative factor of productivity is achieved at the lowest tested fluence of ~ 2 J/cm^2^, with an increase of 26.9 times. Under this condition, the amount of NPs as well as the bubble dynamics are considerably different from those at 10.5 J/cm^2^, thus the shielding behaviour is also different. At 10.5 J/cm^2^, the productivity multiplicative factor is only 7.1 times, despite the highest productivity achieved in the study. This indicates further energy losses and bubble shielding with increasing fluence. The power dependencies of the productivities in the single-beam and multi-beam PLAL are compared in Fig. 2 (d). A productivity increase of 4.4 is obtained at 60 W, i.e., at the maximal power for a single beam and the minimal power for 49 beams. Moreover, Fig. 2 (d) clearly illustrates the DOE-assisted enhancement of the productivity when a high-power laser is used.

**Fig. 3:**
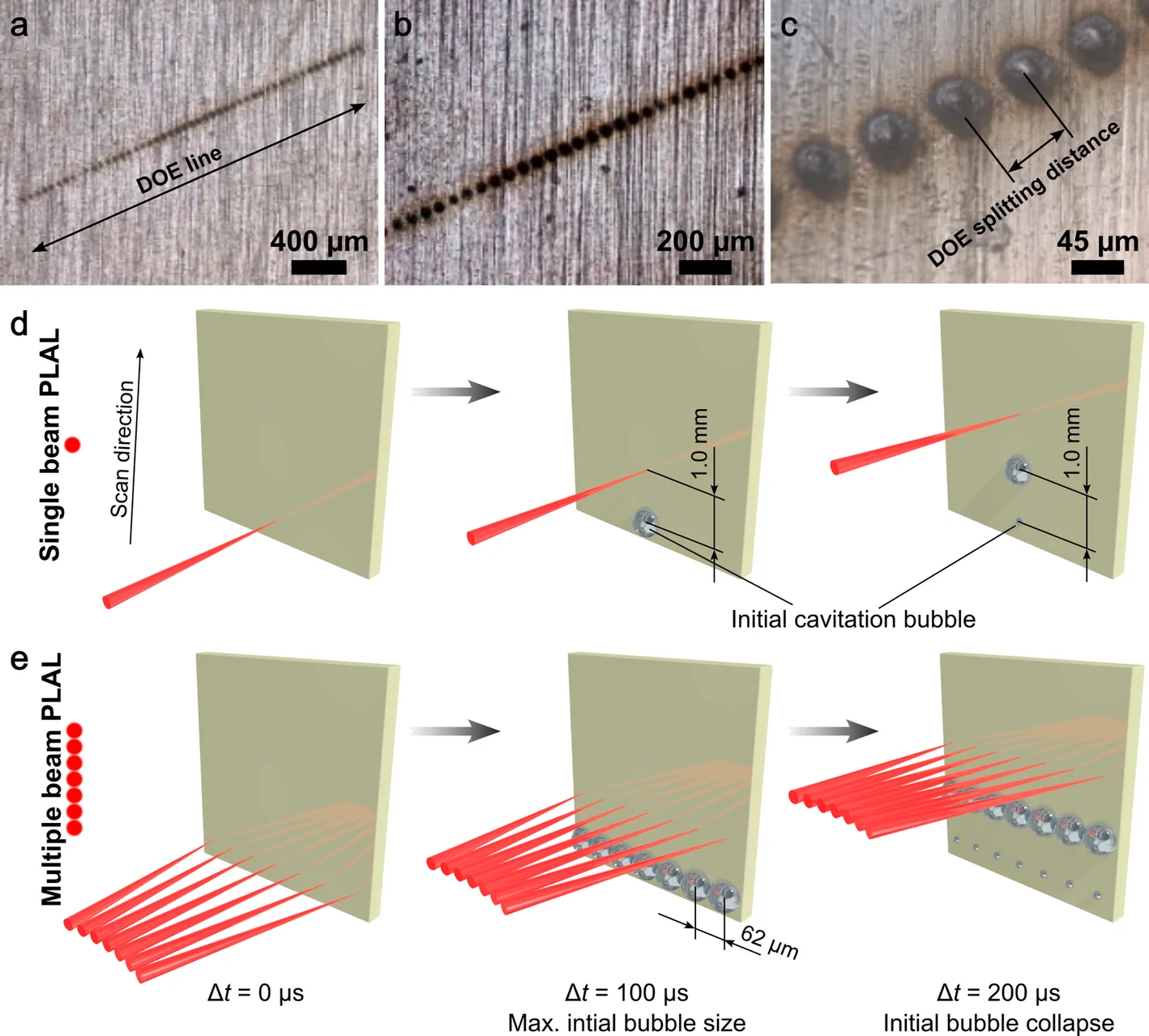
Overview of the different geometrical parameters in single-beam and multi-beam PLAL, (**a**-**c**) optical images illustrating the splitting distance of the neighbouring DOE spots and the total length of the DOE line. **d**-**e** Expected cavitation bubble dynamics in correlation with interpulse time and distance for single-beam (**d**) and multi-beam (**e**) PLAL

Figure 3 illustrates the split-spot array and its relationship with the scanning interpulse distance. The total DOE line with 49 spots at the target position, as well as individual DOE spots, are shown in Figs. 3 (a-c). The splitting distance between two neighbouring spots is measured to be 62 μm, and thus the total length of the DOE line is ~ 3 mm.

For Au ns-PLAL in water and for the considered parameters, the time needed for the cavitation bubble to reach its maximum size is typically ~ 100 µs (which equals to the interpulse time) and ~ 200–300 µs until collapse [33, 34]. The maximum radius of the bubble depends on pulse energy, but it’s typically below 1 mm [33]. While the cavitation bubble dynamics discussed here are interpreted based on established literature under equivalent conditions (ns-PLAL of Au in water), direct spatiotemporal visualisation via high-speed shadowgraphy will be valuable in future studies to precisely capture the complex adjacent bubble interactions during multi-beam PLAL.

Figures 3 (d) and (e) show the expected cavitation bubble evolution in correlation with the interpulse distance and time. The interpulse distance of 1 mm (obtained from a repetition rate of 10 kHz and scanning speed of 10 m/s) guarantees that the neighbouring cavitation bubbles from the successive pulses are positioned far from each other, reducing bubble interaction. This is applicable for both a single beam (Fig. 3 (d)) and multiple beams (Fig. 3 (e)). Moreover, a long enough time between the pulses (100 µs) ensures that, when a cavitation bubble reaches its maximum size and starts to collapse, the following bubble is still in the early growth stage. Thus, temporal and spatial parameters from the laser irradiation minimise cavitation bubble shielding. However, the short DOE splitting distance of 62 μm between the neighbouring spots (Fig, 3(c)) may induce interactions between the simultaneously growing cavitation bubbles.

Since the lifespan of cavitation bubbles is relatively short (~ 200–300 µs), their impact on beam shielding is minimal. Therefore, other factors are expected to induce shielding and affect the productivity behaviour, especially for multi-beam PLAL, when its ablation efficiency (power-specific productivity and the multiplicative factor) decreases with increasing fluence from 2 to 10.5 J/cm^2^.

Persistent bubbles are one important factor that can reduce the ablation efficiency, as they typically remain stationary and adhere to the target [35]. Furthermore, the ejected NPs upon the cavitation bubble collapse can absorb an energy fraction of the subsequent pulses [36, 37]. The NPs can be trapped in a stationary layer within the irradiation region, promoting further shielding [9]. It was shown that both persistent bubbles and NPs may highly shield the beam. Depending on the liquid flow conditions and viscosity, they can shield up to 80% of the incident pulse energy [20].

**Fig. 4:**
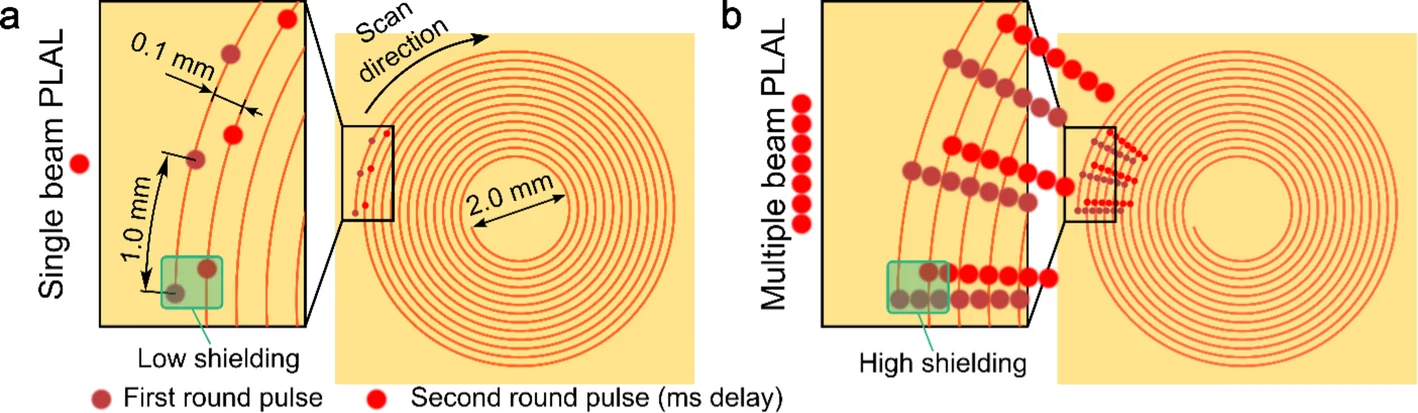
Spiral pattern cyclic and its relation to the spot geometry and interpulse distance for single-beam (**a**) and multi-beam (**b**) PLAL. With indicated spots from two adjacent cyclic turns. In the latter case, the density of the spots within the highlighted green region is higher, resulting in stronger shielding

Figures 4 (a, b) illustrate geometrical parameters of the scanning pattern and their correlation with the interpulse distance and the DOE splitting array. The spiral pattern has 40 cyclic turns with a spiral pitch of 100 μm. The maximum inter-pass time (the time between two spatially adjacent lines on consecutive spiral rounds) is ~ 3214 µs, while the minimum is ~ 643 µs. The choice of the spiral pattern can be explained by its long scanning pattern length of 754 mm and the corresponding scanning time of 0.075 s. Considering the laminar flow with a low speed of 16.7 mm/s, the ability of the liquid flow to remove the NPs away from the irradiation area remains limited. Thus, shielding by the generated NPs and persistent bubbles is expected near the target and within the irradiation area. Note that the persistent bubble lifetime is significantly longer than the inter-pass time between two cycles of the spiral pattern.

While both single beam and multiple beams configurations are expected to form persistent bubbles and cause NP shielding, multi-beam PLAL is expected to induce significantly more persistent bubbles since the number of pulses is 49 times larger. Moreover, its higher productivity means a larger amount of NPs that increase absorption and shielding. Figure 4 illustrates a higher spot density in multi-beam PLAL within a selected region of two spiral cycles. Considering that the DOE line array (2.9 mm) is longer than the spiral pitch (0.1 mm) and that the spiral cycle time is relatively short (643–3214 µs), regions of high shielding by the NPs and persistent bubbles are expected within the irradiation area for the multi-beam PLAL. Furthermore, avalanche ionisation is another factor that can actively decrease ablation efficiency at high fluence regimes. While this phenomenon is much more common during fs pulsed ablation due to the extreme peak intensities involved, it can also occur during high-fluence ns-PLAL. A high concentration of metallic NPs can induce excited free electrons, which can act as seeds, triggering localised breakdown and ionisation of the liquid [38]. This plasma can heavily scatter and absorb the incident beam and reduce the energy delivered to the target, decreasing the power-specific productivity.

Despite the efficiency losses and the decrease in power-specific productivity in multi-beam PLAL, the productivity results obtained in this study remain promising, especially when the economic factors are considered.

The ns laser benefits from its low repetition rate and high pulse energy, which makes it possible to use a DOE with 49 beams and obtain desirable fluence per beam. Compared with the highest productivity reported for Au PLAL, obtained with a ps laser and a high-speed polygon scanner [11], a cost-effective galvanometer scanner was used in this study. The costs of the lasers and their corresponding equipment are illustrated in Table 1. The initial investment cost of a ps laser system is substantially higher than that of an ns laser system, even though both can achieve comparable gram-per-hour productivity. When evaluating economic feasibility, it is essential to consider investment-specific productivity (mg / (h·k€)). Based on this factor, the ns laser is ~ 2.4 times more cost-efficient than the ps laser.

**Table 1:** Economic of ns and ps laser systems used for scaled-up synthesis of NPs. Prices represent initial capital investment costs for the main system components. Operational, maintenance, and lifetime costs are excluded

Specifications	ns laser	ps laser
	High pulse energy, low repetition rate	Low pulse energy, high repetition rate
Laser Price (€, for 500 W)	~ 230,000	~ 550,000
Required equipment for scaled-up production	Standard-speed galvanometer scanner, 1 × 49 DOE	High-speed polygon scanner
Price of the equipment	~ 20,000	~ 150,000
Initial investment price	~ 250,000	~ 700,000
Productivity (mg/hour)	3300 (this work)	3800 (Streubel et al. [9])
Investment-specific productivity (mg/ (hk€))	13.2	5.4

**Fig. 5:**
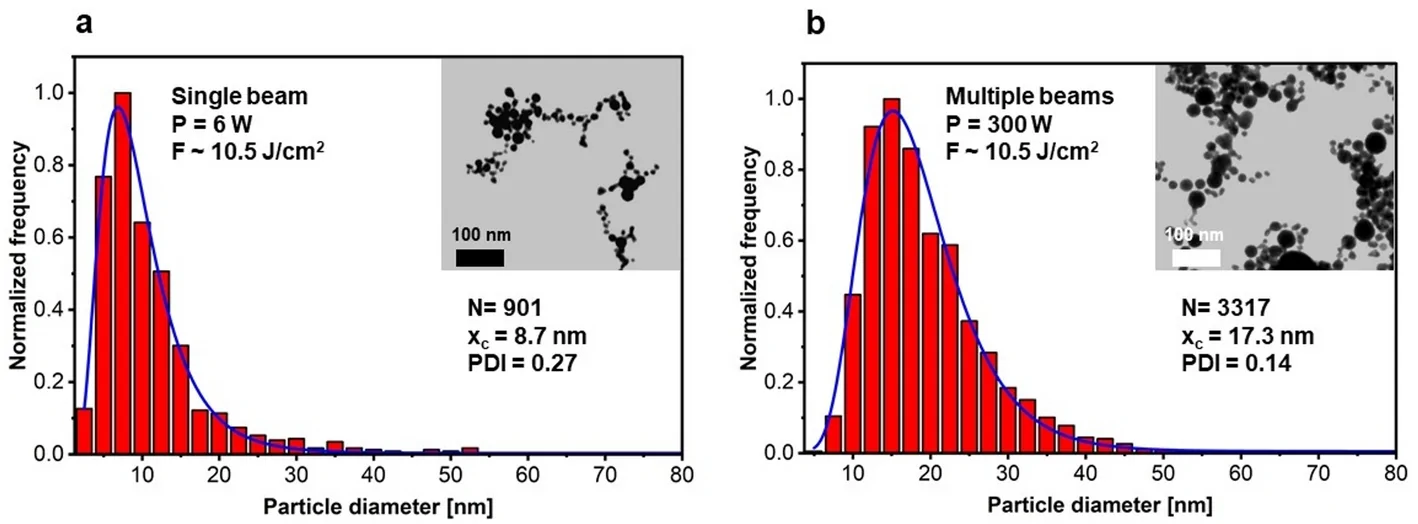
Au NP analysis using STEM for the single-beam (**a**) and multi-beam (**b**) conditions at a fluence of 10.5 J/cm^2^.The number of counted NPs (N), median size (*x*_*c*_), and polydispersity index (PDI) are indicated. Lognormal fittings (lines) were used to calculate *x*_*c*_ and PDI

Figure 5 illustrates the NP analysis using STEM for both single-beam and multi-beam PLAL. In the first case, similar NP size distributions were obtained at different fluences in the range 2–46 J/cm^2^. results in similar NP distributions with comparable values of the median size (*x*_*c*_) and polydispersity index (PDI). Thus, only one representative condition at F_optimal_ = 10.5 J/cm^2^ is illustrated in Fig. 5 (a).

For multi-beam PLAL, NPs produced at a sub-beam fluence of 10.5 J/cm^2^ (i.e., at 300 W input power) were analysed (Fig. 2 (b)) to directly compare the two PLAL conditions at the same fluence per beam. Note also that the highest mass productivity was obtained at 10.5 J/cm^2^ for both regimes.

As seen in Fig. 5, the median NP size increased almost twofold with multi-beam PLAL. This increase indicates different NP growth kinetics during and/or after PLAL. For multiple beams, the short spot separation of 62 μm can induce interaction between the neighbouring cavitation bubbles within the DOE linear array. This can prolong the lifetime of the cavitation bubble and consequently the NP growth phase, leading to the formation of larger NPs [39]. Such an effect was demonstrated by Riahi et al. using a double-beam setup, where the size of Au NPs increased compared to a single beam when the lateral pulse distance between the neighbouring bubbles was set to 75 μm, using a spatially separated double-beam setup [39].

Moreover, since productivity increases by almost an order of magnitude (Fig. 2 (c)), it promotes Ostwald ripening of surface-active Au NPs, and a higher collision frequency can occur during and after PLAL [40].

Other complex factors can affect NP growth during multi-beam PLAL, the simultaneous shockwaves generated across the 1 × 49 array propagate with extreme initial peak pressures in the GPa range, which can induce secondary fragmentation or drive NP to aggregate near the target interface [41, 42]. Given the tight spatial proximity of the sub-beams, these shockwaves undergo complex constructive and destructive interference.

While these proposed mechanisms provide a plausible framework for the observed size shift, detailed spatial mapping and hydrodynamic modelling of multi-beam array interactions remain beyond the scope of this study and warrant future dedicated investigation.

Despite the formation of larger NPs in the multi-beam PLAL, the median size is still within the sub-20 nm range with a negligible fraction of NPs exceeding 80 nm. The size distributions remain reasonably narrow, and the PDI value is even lower than that in the single-beam PLAL. The sub-20 nm NPs retain an outstanding surface-to-volume ratio, and the shift in size does not compromise the functionality of Au NPs in heterogeneous catalysis and biomedical applications [43]. Moreover, if the productivity of the active surface (m^2^/hour) is taken into consideration and calculated from the specific surface area of the median size of both single-beam (8.7 nm) and multi-beam (17.3 nm). This introduces a productivity of 59 m^2^/hour for multi-beam PLAL and 16.4 m^2^/hour for single-beam PLAL, which illustrates that multi-beam PLAL is ~ 3.6 times more efficient than single-beam PLAL in producing active surface area.

It is important to highlight that further studies are needed to clarify the shielding mechanisms affecting the NP generation under multi-beam PLAL.

## Conclusions

Multi-beam PLAL with a high-power ns laser (up to 300 W, pulse energy up to 30 mJ, repetition rate 10 kHz) and a 1 × 49 DOE beam splitter demonstrates excellent PLAL ablation productivity of ~ 3.3 g/h and an outstanding investment-specific productivity of 13.2 mg/ (hk€), which represents a step forward in the use of beam splitting with cost-effective ns lasers. This approach surpasses economically the previous scaled-up approaches that rely on high-power ultrafast lasers. It is shown that the beam-splitting technique allows for the efficient use of the full power of the laser, up to 300 W, whereas, in the case of a single beam, no productivity enhancement is observed at powers above 6 W. The shielding from the cavitation bubble in ns multi-beam PLAL is expected to be rather low, while NPs and persistent bubbles can decrease the ablation efficiency, resulting in reduced power-specific productivity and multiplicative factor, especially at high fluences. Despite the ablation efficiency losses, this approach remains economically effective and unlocks new possibilities to utilise ns lasers for scaled-up synthesis of NPs.

Although the demonstrated throughput of ~ 3.3 g/h represents a significant leap for laser-based synthesis, PLAL production rates are still below the kilogram-per-hour demand for commercial routes. Future efforts combining ultra-high-speed flow, optimised pulse spacing, and more efficient multi-beam spatial configurations will be essential to further decrease energy losses and bridge the gap toward an industrial scale.

## Data Availability

The authors confirm that all data supporting the findings of this study are fully integrated and visually illustrated within the manuscript.
